# Micro Lubrication and Heat Transfer in Wedge-Shaped Channel Slider with Convex Surface Texture Based on Lattice Boltzmann Method

**DOI:** 10.3390/nano14030295

**Published:** 2024-01-31

**Authors:** Jinwei Fang, Xiaori Liu, Tianqi Wang, Zhen Song

**Affiliations:** 1 School of Energy and Environmental Engineering, Hebei University of Technology, Tianjin 300401, China; fangjinwei@baicgroup.com.cn (J.F.); 202121301003@stu.hebut.edu.cn (T.W.); 202121301024@stu.hebut.edu.cn (Z.S.); 2 Beijing Automotive Technology Institute Center Company Limited, Beijing 101300, China

**Keywords:** micro lubrication, lattice Boltzmann method, convex surface texture, temperature distribution, oil film pressure

## Abstract

Hydrodynamic lubrication is widely used between two relatively moving objects, and the effect of fluid flow state and temperature distribution on lubrication performance in wedge-shaped gaps is a popular topic to study. In this paper, the incompressible double-distribution lattice Boltzmann method (LBM) is applied to study the effect of micro convex surface texture on micro lubrication and heat transfer in wedge-shaped channels. By comparing this model with the analytical solution of an infinitely wide wedge slider, the maximum pressure calculated by LBM is 0.1081 MPa, and the maximum pressure calculated by the Reynolds equation is 0.1079 MPa. The error of the maximum pressure is 1.11%, and the Reynolds equation result is slightly smaller. The reason is that the Reynolds equation ignores the influence of fluid inertia force on oil film pressure. The results indicate that the application of LBM can be used to study lubrication problems. Compared with the Reynolds equation, LBM can calculate the velocity field and pressure field in the film thickness direction, and can also observe precise flow field details such as vortices. Three micro convex texture shapes were established to study the effects of different convex textures on micro lubrication and oil film temperature distribution, and the velocity distribution, temperature distribution and oil film pressure along the oil film thickness direction were given. Under the same conditions, comparing the oil film pressure with and without surface texture, the results show that the maximum oil film pressure with surface texture 3 is increased by about 4.34% compared with that without surface texture. The slightly convex texture can increase the hydrodynamic lubrication effect and obtain greater load-bearing capacity, helping to reduce the possibility of contact friction. The results show that the convex surface texture can improve the hydrodynamic lubrication performance, increase the load carrying capacity and reduce the possibility of contact friction, and the convex surface texture can influence the temperature distribution of the oil film. At 3.6 mm in the slider length direction and 7.5 μm in the oil film thickness direction, the temperature of surface texture 1 is 402.64 K, the temperature of surface texture 2 is 403.31 K, and the temperature of surface texture 3 is 403.99 K. The presence of vortices is captured at a high convergence ratio.

## 1. Introduction

Proper surface textures are thought to increase lubrication properties. It is widely believed that surface weaving can store and release lubricant to avoid lubricant deficiency under hydrodynamic and hybrid lubrication and reduce the possibility of surface contact [1]. In addition, the presence of surface textures can store wear debris and thus reduce wear [2]. There have been many experimental and theoretical studies on the micro surface texture of friction pairs. Li et al. [3] designed kite-shaped micro-textures to improve the lubrication performance of tribological systems under hydrodynamic lubrication. Wang et al. [4] proposed a prediction model of the friction coefficient and validated this for polished and microscopic, mesoscopic, and macroscopic textured surfaces. Shen et al. [5] investigated the lubrication performance of chevron textured surfaces under hydrodynamic lubrication conditions by numerical simulation. The results show that the geometric parameters such as depth, length, width, and angle of the chevron textures have significant effects on the lubrication performance. There have been many experimental and theoretical studies on the micro surface texture of surfaces. Microscopic convex texture has an optimization effect on lubrication and friction in many cases. Guo et al. [6] studied the water-lubricated stern tube bearing, and through tests, showed that convex micro-texture could reduce wear, and convex texture was the most effective design to improve wear performance at low sliding speeds of 0.063 m/s.

The heat transfer of lubrication has not been considered by the above-mentioned studies. Gu et al. [7] used a thermal mixing lubrication model and an isothermal model, which were used to investigate the effect of different inlet oil temperatures on the lubrication performance of micro-textured ring/liner conjunction. The results show that when the boundary temperature is low, the clearance between the piston ring and cylinder liner becomes larger, which is not conducive to heat transfer. The convex surface texture has been found to be an important surface texture. Pei et al. [8] investigated the effect of micro surface texture on the performance of floating ring bearings using a multiscale finite element method and showed that the convex texture had a more significant effect on the bearing performance than the concave texture, and that the surface texture could increase the local pressure distribution and side leakage, thus reducing the temperature rise of the film. Ruan et al. [9] studied gear meshing with consideration of the influence of the contact interface of micro-texture and frictional thermal load; it was found that the influence of the micro-texture on gear dynamic response was extremely restricted by the transient contact regularity of the meshing gear surface.

For many friction pairs with a high heat load, heat transfer is important in lubrication research, and the temperature of the oil film has a significant impact on lubrication performance [10,11,12]. There are several ways to solve lubrication and lubricant film temperature distribution, such as the Reynolds equation and the Navier–Stokes (N-S) equation [13,14,15]. The oil film pressure and temperature distribution can be solved by combining the Reynolds equation and the energy equation, but the Reynolds equation cannot calculate the pressure and velocity distribution in the direction of the oil film thickness. Although the pressure distribution and velocity distribution of the oil film can be solved, the N-S equation is based on the continuity assumption and requires high mesh quality.

The lattice Boltzmann method (LBM) is a method of computational fluid dynamics based on Boltzmann transport equations that has been successfully applied to computational fluid flow and heat transfer problems; LBM can calculate the lubrication whether the fluid is continuous or discontinuous medium [16,17,18,19]. Brenner et al. [20] used LBM to analyze the effect of surface texture and roughness on shear force and pressure. The results show that the application of LBM can improve the tribological modeling capability by fully considering the geometric surface features. Jiao et al. [21] used LBM to analyze linear, parabolic and harmonic wedge models, giving the velocity distribution of the flow field and the corresponding bearing capacity, and also studied the pressure distribution of different types of wedges.

The study of wedge gap lubrication is the basis for studying the lubrication mechanism. There are many mechanical components with wedge gap such as thrust bearings, mechanical face seals, piston ring systems, traditional metal processing field and sliding guideways with wedging action [22,23,24,25,26]. There are still the following issues that need to be further improved when using LBM to calculate lubrication: (1) When dealing with irregular shape boundaries, the problem of non-overlapping between lattice points and solid boundaries needs to be solved to restore geometric integrity. Therefore, when using LBM for lubrication, the problem of lattice points and solid boundaries not overlapping needs to be further addressed [21]. (2) Previous studies often obtained results in dimensionless units without unit conversion, which cannot be applied to engineering calculations [21,27]. (3) The Reynolds equation can calculate the lubricating oil film pressure of the frictional pair, but it cannot provide the velocity and temperature distribution in the oil film in the direction of the oil film thickness. Gu et al. [7] combined the Reynolds equation with the Navier–Stokes equation to derive the velocity distribution; in the existing LBM lubrication calculations, further clarification is needed on the distribution of lubricating oil flow velocity in the lubrication gap, and further research is needed on the impact of changes in micro-texture structural parameters on lubricating oil flow.

As discussed above, micro convex texture has an optimization effect on lubrication and friction in many cases. Using LBM to obtain the velocity and temperature distribution in the oil film in the direction of the oil film thickness is helpful for texture design and can also provide a more comprehensive understanding of its lubrication and heat transfer mechanisms. The micro lubrication and heat transfer numerical model in wedge-shaped channel sliders with a convex surface texture based on the lattice Boltzmann method should be established accurately. In this paper, three convex surface textures were established, and the incompressible double-distribution LBM was utilized to calculate the micro lubrication and heat transfer in wedge-shaped channel sliders. The wedge shape, micro convex texture shape and interface velocity boundary were restored with the methods of non-equilibrium extrapolation and spatial interpolation. Spatial step size was chosen to restore the true physical dimensions and units. The effect of different micro convex surface textures on velocity distribution, streamline linearity and temperature distribution, which Reynolds equation could not provide, were investigated. The lubrication performance of sliders with and without textured surfaces was compared to provide a basis for studying the effect of textured surfaces on micro lubrication and oil film temperature.

## 2. The Physical Model

**Figure 1:**
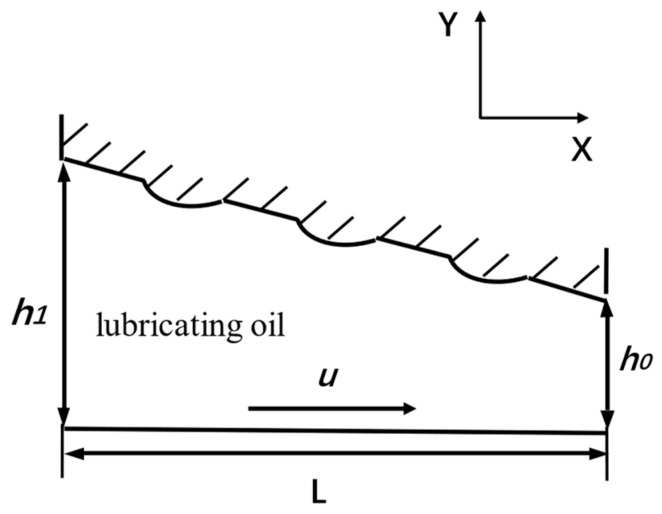
Model of a wedge with micro surface texture.

The model of the wedge slider is shown in Figure 1; the bottom edge of the wedge moves to the right with speed u; the length of the wedge is L; the micro oil film thickness of the left inlet is $h_{1}$; the micro oil film thickness of the right outlet is $h_{0}$; the ratio of inlet and outlet oil film thickness is K,$\;K=\frac{h_{1}}{h_{0}}$. The tilt angle of the wedge is determined by the value of K, and the equation of the slope is as follows

(1) $$h=-\frac{h_{1}-h_{0}}{L}x+h_{1}$$

Based on the wedge slider, the sine function is applied to model the micro surface texture, and the sine function is as follows

(2) $$y=A\sin\left(\omega x\right)$$

where A is the amplitude, and ω is the scaling factor of the transverse coordinate. The values that take the sine function less than 0 are applied to the modeling of the surface texture. The surface texture is obtained by adding the value of the sine function less than 0 to the slope equation

(3) $$H=-\frac{h_{1}-h_{0}}{L}x+h_{1}+y$$

The lubricant can reduce friction and also play the role of auxiliary cooling. This paper does not aim at specific friction pairs in actual engineering cases, and takes common mineral lubricating oil as the object. Here, the lubricant data of 0W-20 lubricating oil are taken as an example to provide reference for similar situations. The data for the lubricant are given in Table 1.

## 3. Lattice Boltzmann Method

### 3.1. Lattice Boltzmann Equation of Flow Field

**Figure 2:**
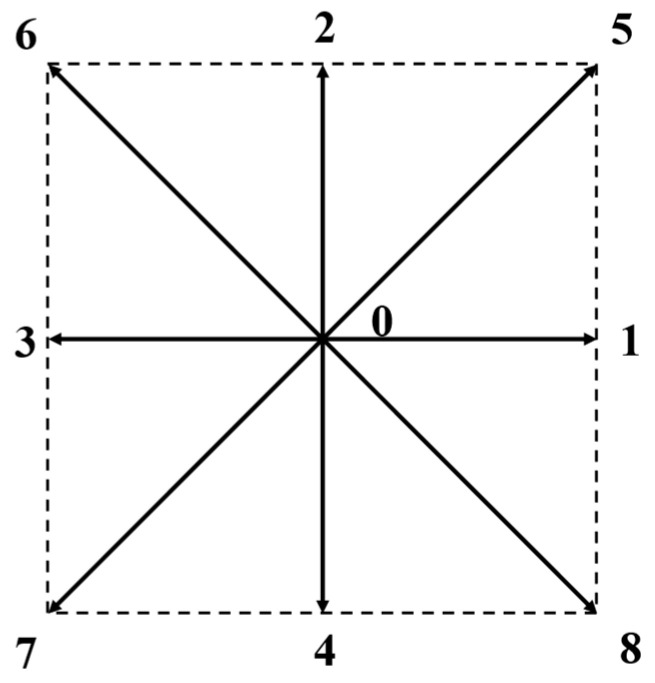
The two-dimensional, nine-velocity lattice.

The predecessor of LBM was the lattice gas automata (LGA), which is able to obtain continuous momentum based on particle interactions [28,29]. LBM utilizes the fact that the collective behavior of many microscopic particles underlies the macroscopic dynamics of fluids, which is insensitive to the microscopic details of the phenomena exhibited by individual molecules [30]. In LBM, the mean behavior of agglomerations of particles are studied using probability distribution functions. In the standard LBM, the particle distribution functions are discretized in space on a regular cubic lattice, where particles travel with their discrete velocity from one lattice site to another in one time step [31]. Particle collisions are simulated by calculating the particle distribution function at each position through the operator Ω. Dealing with the collision operator is the challenge of solving the Boltzmann equation, since the macroscopic properties of the fluid are the statistical results of the microscopic motion of the fluid particles, the collision process of the particles has little effect on the statistical results. The most widely used collision operator that can recover the Navier–Stokes equation is the Bhatnagar–Gross–Krook collision operator [32]. Under the BGK approximation, the lattice Boltzmann equation for the flow field takes the form:

(4) $$f_{i}(x+ce_{i}\Delta t,t+\Delta t)-f_{i}\left(x,t\right)=-\frac{1}{\tau_{f}}\left[f_{i}(x,t)-f_{i}^{eq}(x,t)\right]$$

where $\tau_{f}$ represents the time required for the particle distribution function to reach equilibrium and is related to the kinetic viscosity υ. The kinetic viscosity $\upsilon =c_{s}^{2}\left(\tau_{f}-0.5\right)$, $\tau_{f}>0.5$ to ensure a positive viscosity. The two-dimensional lattice with nine velocity directions is applied to our study in this paper. Figure 2 shows a two-dimensional lattice. It has nine velocity vectors, with the central particle speed equal to 0, and the other velocity vectors along the 1–8 direction.

The direction of particle motion velocity, $e_{i}$, can be expressed as:

(5) $$e_{i}=\left\{\begin{array}{c} (0,0),\;\;\;\;\;\;\;\;\;\;\;\;\;\;\;\;\;\;\;\;i=0\\ \left(\cos\left[\frac{\pi }{2}(i-1)\right],\sin\left[\frac{\pi }{2}(i-1)\right]\right),\;\;\;\;\;\;\;\;\;\;i=1,2,3,4\\ \sqrt{2}\left(\cos\left(\frac{\pi }{2}(i-5)+\frac{\pi }{4}\right),\sin\left(\frac{\pi }{2}(i-5)+\frac{\pi }{4}\right)\right),\;\;\;\;\;i=5,6,7,8 \end{array}\right.$$

where the equilibrium distribution function $f_{i}^{eq}$ is given by the macroscopic volume density ρ and the velocity u:

(6) $$f_{i}^{eq}=w_{i}\rho \left[1+3\frac{e_{i}\cdot u}{c}+\frac{9}{2}\frac{(e_{i}\cdot u{)}^{2}}{c_{\;}^{2}}-\frac{3}{2}\frac{u\cdot u}{c_{\;}^{2}}\right]$$

$w_{i}$ is the weight factor related to the velocity direction, and the weight factors corresponding to different velocity directions are expressed as: $w_{0}=4/9$, $w_{1,2,3,4}=1/9$, $w_{5,6,7,8}=1/36$. The lattice velocity c is related to the spatial step and the time step, where $c=\frac{\Delta x}{\Delta t}$. In general, it is customary to nondimensionalize the spatial step and the time step as Δx=Δt=1, which gives c=1. The macroscopic volume velocity u and density ρ of the fluid can be obtained from the zero-order and first-order moments of the distribution function. The flow velocity u are calculated by

(7) $$\rho u=\sum_{i}ce_{i}f_{i}$$

The density ρ is calculated by

(8) $$\rho =\sum_{i}f_{i}$$

The LBM equation evolution process is divided into two processes: collision and streaming. The collision process is local and involves only the local node, which can be expressed by the distribution function as

(9) $$f_{i}^{+}(x,t+\Delta t)=(1-\frac{1}{\tau_{f}})f_{i}(x,t)+\frac{1}{\tau_{f}}f_{i}^{eq}(x,t)$$

The streaming process is related to the neighboring particles and is expressed through the distribution function as

(10) $$f_{i}(x+ce_{i}\Delta t,t+\Delta t)=f_{i}^{+}(x,t)$$

Due to the fact that the LBM method is not a fully incompressible model, errors can occur in the calculation process for incompressible fluids. In this paper, an incompressible LBGK model proposed by He et al. is used [33]. In He’s incompressible model, the pressure p is used as an independent variable and the new equilibrium distribution function obtained is

(11) $$f_{i}^{eq}=w_{i}\left\{\rho +\rho_{0}\left[3\frac{e_{i}\cdot u}{c}+\right.\right.\frac{9}{2}\frac{(e_{i}\cdot u{)}^{2}}{c^{2}}-\left.\left.\frac{3}{2}\frac{u\cdot u}{c^{2}}\right]\right\}$$

For incompressible fluids, the density of the fluid is approximated by the constant $\rho_{0}$. In the incompressible model, the velocity is calculated from

(12) $$\rho_{0}u=\sum_{i}ce_{i}f_{i}$$

### 3.2. Lattice Boltzmann Equation of Temperature

The double-distribution function (DDF) models are considered to be the most successful LBM framework for solving thermal problems [34]. In this model, two distribution functions are used, one for the density distribution function and the other for the internal energy distribution function [35]. The lattice Boltzmann equation for the temperature field is given by

(13) $$g_{i}(x+ce_{i}\Delta t,t+\Delta t)-g_{i}\left(x,t\right)=-\frac{1}{\tau_{g}}\left[g_{i}(x,t)-g_{i}^{eq}(x,t)\right]$$

In this case, the distribution function of the temperature field is given by $g_{i}$. Where $\tau_{g}$ is the relaxation time associated with the thermal diffusion coefficient α, where the thermal diffusion coefficient can be given as $\alpha =c_{s}^{2}\left(\tau_{g}-0.5\right)$. $\tau_{g}>0.5$ ensures that the thermal diffusion coefficient is greater than 0. The equilibrium distribution function of the temperature field is $g_{i}^{eq}$, which can be given the following equation

(14) $$g_{i}^{eq}=w_{i}T\left(1+\frac{e_{i}\cdot u}{c_{s}^{2}}\right)$$

where T denotes the fluid temperature, which is calculated from the following equation

(15) $$T=\sum_{i}g_{i}$$

The evolution equation of the temperature field is the same as the flow field, divided into two processes of collision and streaming, the collision process is

(16) $$g_{i}^{+}(x,t+\Delta t)=(1-\frac{1}{\tau_{g}})g_{i}(x,t)+\frac{1}{\tau_{g}}g_{i}^{eq}(x,t)$$

The streaming process is

(17) $$g_{i}(x+ce_{i}\Delta t,t+\Delta t)=g_{i}^{+}(x,t)$$

### 3.3. Boundary Conditions

**Figure 3:**
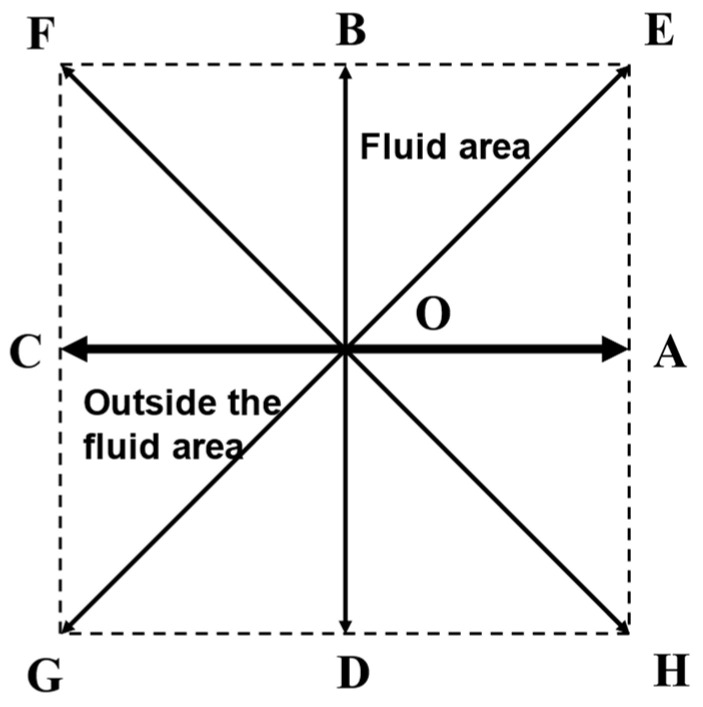
Lattice points at the boundary.

Velocity and pressure boundary conditions are often required in lubrication. Guo’s proposed method based on non-equilibrium partial extrapolation to deal with velocity and pressure boundaries is applied in this study [36]. The lattice points at the boundary are shown in Figure 3.

C, O and A are the boundary points, F, B and E are located in the fluid region, and G, D and H are the parts outside the fluid region. $f_{i}$ at point O is decomposed into an equilibrium distribution function $f_{i}^{eq}$ and nonequilibrium distribution function $f_{i}^{neq}$. Only one macroscopic variable velocity or density is unknown at the boundary node O. For the velocity boundary, the unknown parameter $\rho_{O}$ is replaced by the neighboring node $\rho_{B}$, the $u_{bc}$ is the known boundary velocity, and the equilibrium state distribution function can be written as

(18) $$f_{i}^{eq}=w_{i}\left\{\rho_{B}+\rho_{0}\left[3\frac{e_{i}\cdot u_{bc}}{c}+\frac{9}{2}\frac{(e_{i}\cdot u_{bc}{)}^{2}}{c^{2}}-\right.\right.\left.\left.\frac{3}{2}\frac{u_{bc}\cdot u_{bc}}{c^{2}}\right]\right\}$$

For the pressure boundary, the unknown parameter $u_{O}$ is replaced by the neighboring node $u_{B}$, the pressure *p* is a known boundary pressure, and the equilibrium state distribution function can be written as

(19) $$f_{i}^{eq}=w_{i}\left\{\frac{p_{bc}}{c_{s}^{2}}+\rho_{0}\left[3\frac{e_{i}\cdot u_{B}}{c}+\frac{9}{2}\frac{(e_{i}\cdot u_{B}{)}^{2}}{c^{2}}\right.\right.\left.\left.-\frac{3}{2}\frac{u_{B}\cdot u_{B}}{c^{2}}\right]\right\}$$

For the non-equilibrium part at the boundary node O can be replaced by the non-equilibrium part at the adjacent node B

(20) $$f_{i}^{neq}(O,t)\approx f_{i}^{neq}(B,t)=f_{i}(B,t)-f_{i}^{eq}(B,t)$$

**Figure 4:**
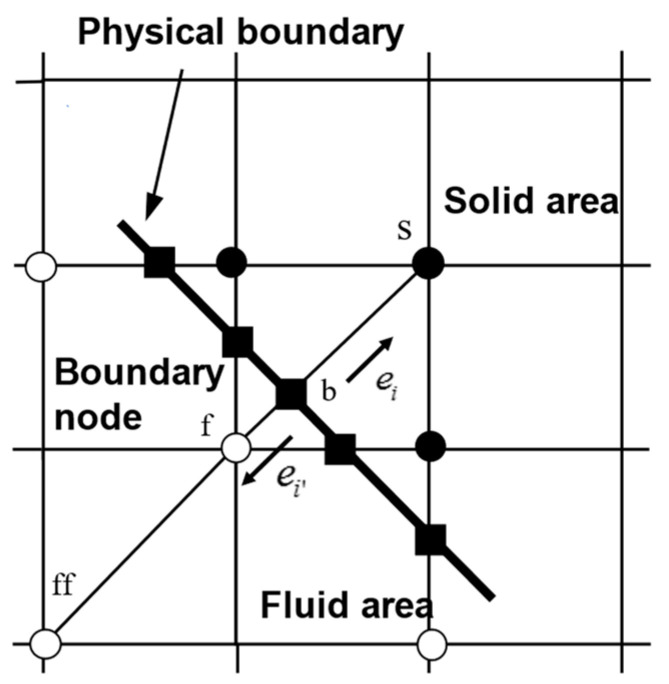
Types of boundary nodes.

To solve the problem that the upper surface lattice nodes of the wedge slider do not overlap with the physical boundary, a method based on non-equilibrium extrapolation and spatial interpolation proposed by Guo et al. was applied to this study [37]. The method has second-order accuracy in both time and space. As shown in Figure 4, the curved boundary is used to distinguish the fluid region from the solid region. The initial direction of the particle is $e_{i}$ and will move in the opposite direction $e_{i’}$ when it encounters a physical boundary.

The nodes in the fluid region are $x_{f}$, the physical boundary node $x_{b}$ and the solid node $x_{s}$. The link between the fluid node $x_{f}$ and the solid node $x_{s}$ intersects the physical boundary at the boundary node $x_{b}$. The percentage of fluid area is denoted by q

(21) $$0\leq q=\frac{\left|x_{f}-x_{b}\right|}{\left|x_{f}-x_{s}\right|}\leq 1$$

Decompose $f_{i}^{+}(x_{s},t)$ into an equilibrium part $f_{i}^{eq}(x_{s},t)$ and a nonequilibrium part $f_{i}^{neq}(x_{s},t)$. The equilibrium distribution function $f_{i}^{eq}(x_{s},t)$ is defined as follows

(22) $$f_{i}^{eq}=w_{i}\left\{\rho_{s}+\rho_{0}\left[3\frac{e_{i}\cdot u_{s}}{c}+\right.\right.\frac{9}{2}\frac{(e_{i}\cdot u_{s}{)}^{2}}{c^{2}}-\left.\left.\frac{3}{2}\frac{u_{s}\cdot u_{s}}{c^{2}}\right]\right\}$$

The unknown density at the solid node $\rho_{s}$ is replaced by the adjacent node $\rho_{f}$. The LBM can be interpreted as a special discrete form of the Boltzmann equation; therefore, it is feasible to determine by interpolation of the segmentation function

(23) $$u_{s}=\left\{\begin{array}{c} (u_{b}+(q-1)u_{f})/q,\;\;\;\;\;\;\;\;\;\;\;\;\;\;\;\;\;\;\;\;\;\;\;\;\;\;\;\;\;\;\;\;\;\;\;\;\;\;\;\;\;\;\;\;\;\;\;\;\;\;\;\;\;\;q\geq 0.75\\ (u_{b}+(q-1)u_{f})+(1-q)\left[2u_{b}+\left(q-1\right)u_{ff}\right]/(1+q),\;\;\;\;\;q<0.75\; \end{array}\right.$$

The nonequilibrium distribution function $f_{i}^{neq}(x_{s},t)$ is defined as follows

(24) $$f_{i}^{neq}=\left\{\begin{array}{c} f_{i}\left(x_{f},t\right)-f_{i}^{eq}\left(x_{f},t\right),\;\;\;\;\;\;\;\;\;\;\;\;\;\;\;\;\;\;\;\;\;\;\;\;\;\;\;\;\;\;\;\;\;\;\;\;\;\;\;\;\;\;\;\;\;\;\;\;\;\;\;\;\;\;\;\;\;\;\;\;q\geq 0.75\\ q\left[f_{i}\left(x_{f},t\right)-f_{i}^{eq}\left(x_{f},t\right)\right]+(1-q)\left[f_{i}\left(x_{ff},t\right)-f_{i}^{eq}\left(x_{ff},t\right)\right],\;\;q<0.75 \end{array}\right.$$

For treating temperature fields with curved boundaries, the methodology of study [38,39] was used in this paper. The distribution function $g_{i’}(x_{s},t)$ of temperature can be decomposed into equilibrium distribution function $g_{i’}^{eq}(x_{s},t)$ and nonequilibrium distribution function $g_{i’}^{neq}(x_{s},t)$

(25) $$g_{i’}(x_{s},t)=g_{i’}^{eq}(x_{s},t)+g_{i’}^{neq}(x_{s},t)$$

where $e_{i}=-e_{i’}$, brought into the evolution equation for the temperature field

(26) $$g_{i’}(x_{s},t)=g_{i’}^{eq}(x_{s},t)+\left(1-\frac{1}{\tau_{g}}\right)g_{i’}^{neq}(x_{s},t)$$

If you need to calculate $g_{i’}(x_{s},t)$, you first need to know $g_{i’}^{eq}(x_{s},t)$ and $g_{i’}^{neq}(x_{s},t)$. The equilibrium distribution function is calculated as follows

(27) $$g_{i’}^{eq}=w_{i’}T_{s}\left(1+\frac{e_{i’}\cdot u_{s}}{c_{s}^{2}}\right)$$

The formula for calculating the velocity $u_{s}$ at the curved boundary of the temperature field is the same as for the velocity field. Where $T_{s}$ is classified according to the value of q,

(28) $$T_{s}=\left\{\begin{array}{c} (T_{b}+(q-1)T_{f})/q,\;\;\;\;\;\;\;\;\;\;\;\;\;\;\;\;\;\;\;\;\;\;\;\;\;\;\;\;\;\;\;\;\;\;\;\;\;\;\;\;\;\;\;\;\;\;\;\;\;\;\;\;\;\;q\geq 0.75\\ (T_{b}+(q-1)T_{f})+(1-q)\left[2T_{b}+\left(q-1\right)T_{ff}\right]/(1+q),\;\;\;\;\;q<0.75\; \end{array}\right.$$

The non-equilibrium distribution function of the equation is defined as

(29) $$g_{i}^{neq}=\left\{\begin{array}{c} g_{i}\left(x_{f},t\right)-g_{i}^{eq}\left(x_{f},t\right),\;\;\;\;\;\;\;\;\;\;\;\;\;\;\;\;\;\;\;\;\;\;\;\;\;\;\;\;\;\;\;\;\;\;\;\;\;\;\;\;\;\;\;\;\;\;\;\;\;\;\;\;\;\;\;\;\;\;\;\;q\geq 0.75\\ q\left[g_{i}\left(x_{f},t\right)-g_{i}^{eq}\left(x_{f},t\right)\right]+(1-q)\left[g_{i}\left(x_{ff},t\right)-g_{i}^{eq}\left(x_{ff},t\right)\right],\;\;q<0.75 \end{array}\right.$$

**Figure 5:**
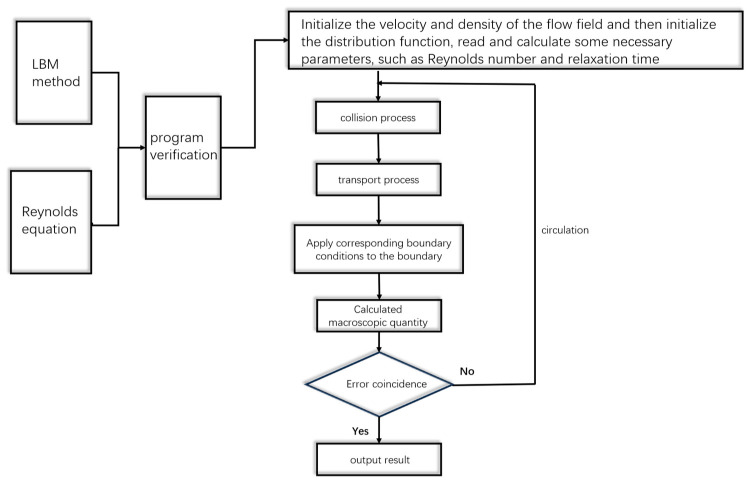
The LBM calculation flow chart.

The LBM calculation flow chart is shown in the Figure 5.

## 4. Validation

**Figure 6:**
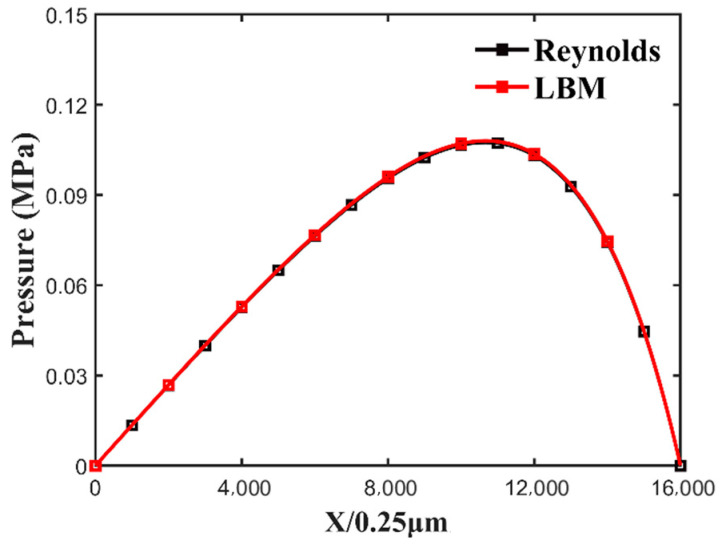
Comparison of LBM and Reynolds equation results.

An infinitely wide wedge slider as shown in Figure 6 is used to verify the accuracy of LBM. The calculation model in this study is programmed using C++. For solving the incompressible steady-state infinitely wide slider, the Reynolds equation can be reduced to a one-dimensional ordinary differential equation as follows

(30) $$\frac{d}{dx}\left(\frac{h^{3}}{\mu }\frac{dp}{dx}\right)=6u\frac{dh}{dx}$$

where p is the oil film pressure, h is the oil film thickness, μ is the dynamic viscosity, and u is the kinematic boundary velocity. The parameters needed for modeling are given in Table 2.

In this study, Reynolds number (*Re*) equality is used to satisfy the similarity of the two physical systems. The inlet oil film thickness was chosen as the characteristic length.

(31) Re⁡=ρulμ

where l is the characteristic length.

In the calculation of the wedge slider, left and right is the pressure boundary of equal pressure. The results of the pressure distribution comparison between the two methods are shown in Figure 6. The maximum pressure calculated by LBM is 0.1081 MPa, the maximum pressure calculated by the Reynolds equation is 0.1079 MPa, and the error of the maximum pressure is only 1.11%. The reason for the slightly smaller result for the Reynolds equation is that the Reynolds equation ignores the effect of fluid inertia forces on the oil film pressure. Numerical results show that the application of LBM can be used to study lubrication problems, and this numerical method is simple and accurate.

## 5. Effect of Surface Texture on Lubrication and Heat Transfer

### 5.1. Setting of Surface Texture and Boundary Conditions

**Figure 7:**
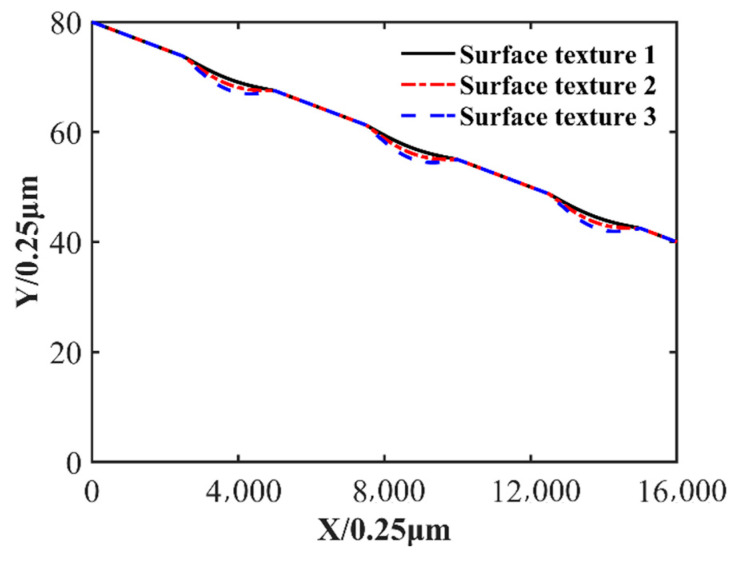
Diagram of the surface textures.

For LBM modeling, the number of lattices in the slider length direction is set to 16,000. In the second part of this paper, when the validity of the Reynolds equation is verified, the verification result is that when the number of grids is 16,000 × 80, the maximum pressure error is 1.11%, which proves that the fluid can be accurately calculated. A 16,000 × 80 grid was also used in subsequent calculations. In this paper, the wedge slider length is 4 mm, and the length of each mesh is 0.25 μm = 4 mm/16,000. When applying the LBM calculation, three different surface textures are built. Three different surface textures are shown in Figure 7. The surface texture is determined by Equations (1)–(3). The parameters needed for Equation (2) are given in Table 3. The inlet and outlet pressure of the slider is set to 0, the velocity is set to u=4 m/s, the inlet temperature is set to 363 K, the outlet temperature uses the fully developed boundary, the upper surface temperature of the wedge is set to 423 K, and the lower surface temperature of the wedge is set to 363 K.

### 5.2. Calculation Results

**Figure 8:**
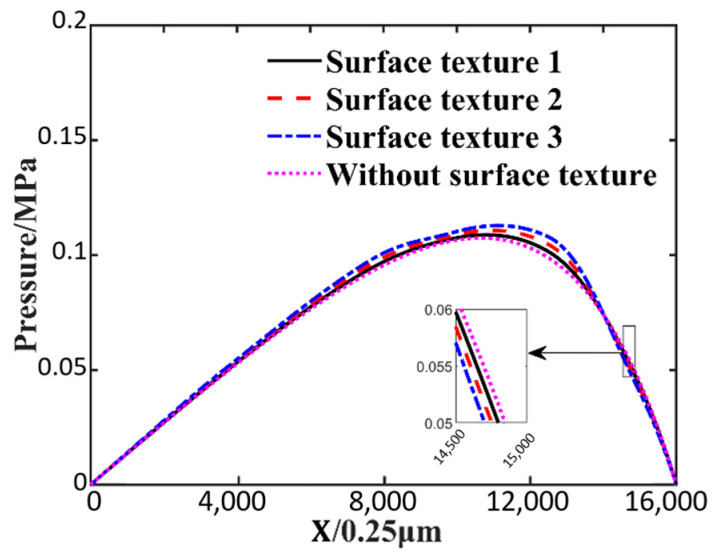
Effect of different surface textures on oil film pressure distribution.

**Figure 9:**
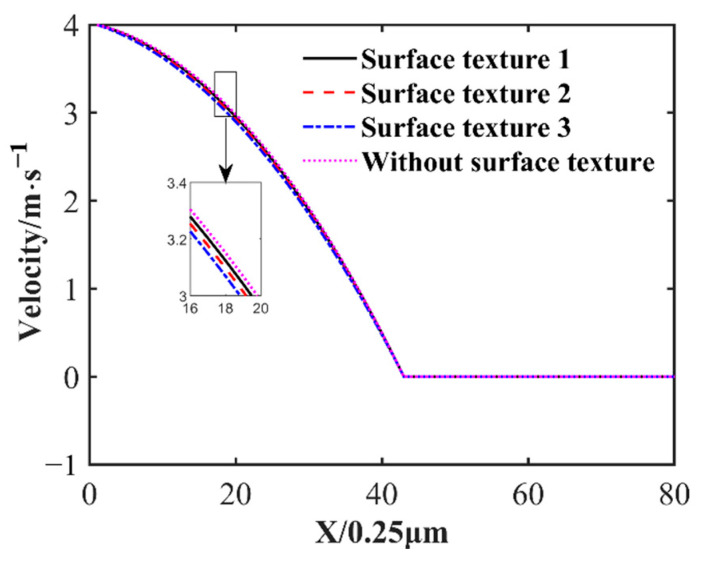
Velocity distribution in the direction of oil film thickness at 3.8 mm in the slider length direction near the outlet.

The effect of three different surface textures on the oil film pressure distribution is depicted in Figure 8. In this figure, it can be seen that a suitable surface texture can increase the maximum oil film pressure. The maximum oil film pressure of surface texture 3 is 0.1128 MPa. The maximum oil film pressure without surface texture is 0.1081 MPa. Surface texture 3 increased the maximum oil film pressure by about 4.34% compared to without surface texture. The higher the convex texture in the middle part of the slider, the smaller the gap between the slider and the greater the pressure. At the outlet position, the influence of flow velocity dominates. The velocity of the outlet position in ascending order is texture 3, texture 2, texture 1 and without texture. The convex texture causes more flow loss and results in a smaller velocity at the outlet position. At the outlet position, the pressure of the three kinds of oil film with surface texture is lower than that of the oil film without surface texture, so the bearing capacity of the oil film with surface texture is lower. As Figure 9 demonstrates the velocity distribution in the direction of oil film thickness at 3.8 mm in the slider length direction near the outlet, the maximum pressure of the oil film without surface texture near the outlet is due to the maximum velocity of the oil film without surface texture.

**Figure 10:**
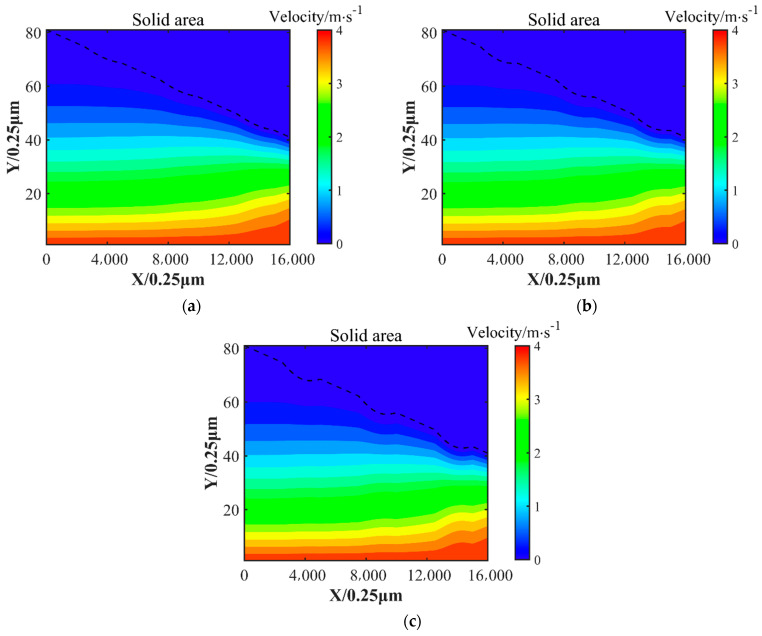
The velocity distribution of the oil film. (**a**) Velocity distribution of oil film on surface texture 1; (**b**) velocity distribution of oil film on surface texture 2; (**c**) velocity distribution of oil film on surface texture 3. The dashed line is the slider profile.

The distribution of the oil film velocity is given in Figure 10. Overall, the lubricating oil flowed in the direction of the outlet position; due to the wedge gap, the fluid flow process was squeezed, and the proportion of fast-flowing fluid gradually increased, in accordance with the fluid flow theory.

**Figure 11:**
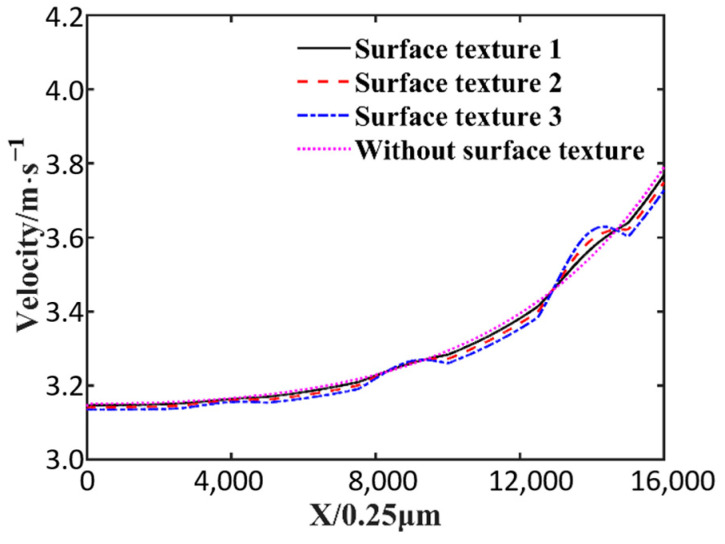
Speed curves at oil film thickness of 2.5 μm.

Figure 11 shows the speed curves at an oil film thickness of 2.5 μm. As shown in the figure, the presence of surface texture can increase the local oil film velocity, and the local velocity increases more significantly as the wedge gap decreases. The largest local velocity increase is at the 3.55 mm position along the *x*-axis, with a 0.50% increase in surface texture 1, a 1.05% increase in surface texture 2, and a 1.61% increase in surface texture 3. The presence of surface texture increases local the lubricating oil velocity but causes a reduction in velocity elsewhere. Due to the surface texture, the cross section decreases and the fluid velocity increases. But at the same time, the presence of surface texture leads to local resistance, so after texture, the fluid speed is smaller than without texture.

**Figure 12:**
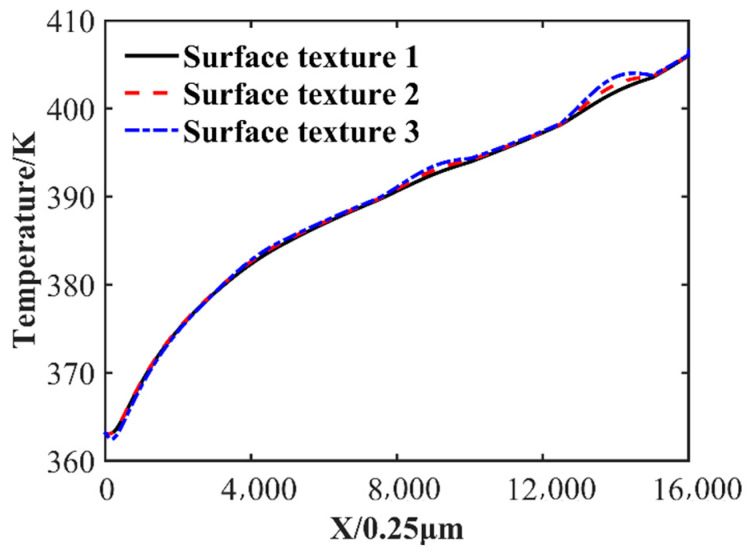
The temperature distribution at the position where the oil film thickness is equal to 7.5 μm.

The temperature distribution at the position where the oil film thickness is equal to 7.5 μm is shown in Figure 12. The presence of surface texture increases local heat transfer. The temperature of surface texture 1 is 402.64 K, that of surface texture 2 is 403.31 K, and that of surface texture 3 is 403.99 K at 3.6 mm in the slider length direction. On the one hand, the heat transfer area is increased due to the presence of the surface texture, and on the other hand, the surface texture protrudes inward, and the distance from the bottom boundary is reduced, which may help the lubricant to dissipate heat.

**Figure 13:**
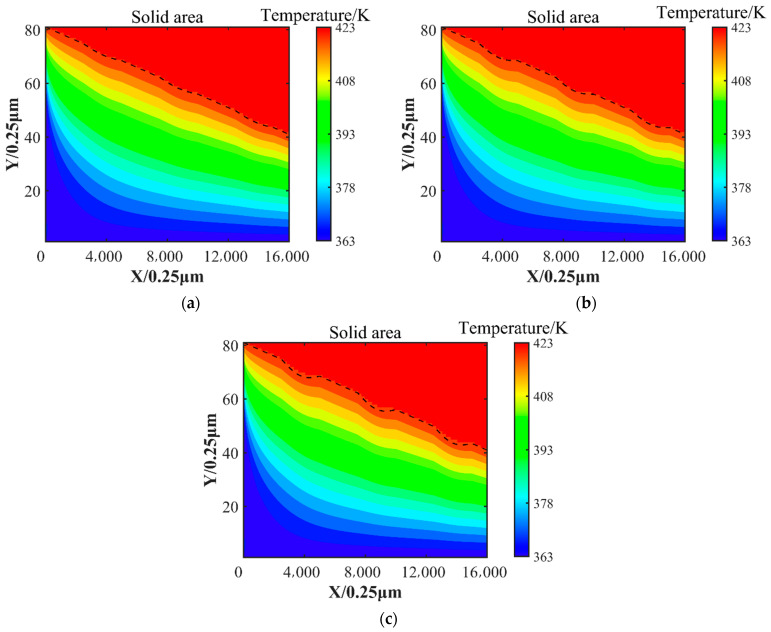
The temperature distribution of the oil film. (**a**) The temperature distribution of surface texture 1; (**b**) the temperature distribution of surface texture 2; (**c**) the temperature distribution of surface texture 3. The dashed line is the slider profile.

The oil film temperature distribution cloud is shown in Figure 13. It can be seen that the temperature gradually increases along the oil film thickness direction. There is a large variation in the oil film temperature distribution at the inlet. As shown in Figure 12, compared with surface texture 1 and surface texture 2, the fluid velocity near the texture is larger, the convection capacity is stronger, and the heat transfer is larger.

**Figure 14:**
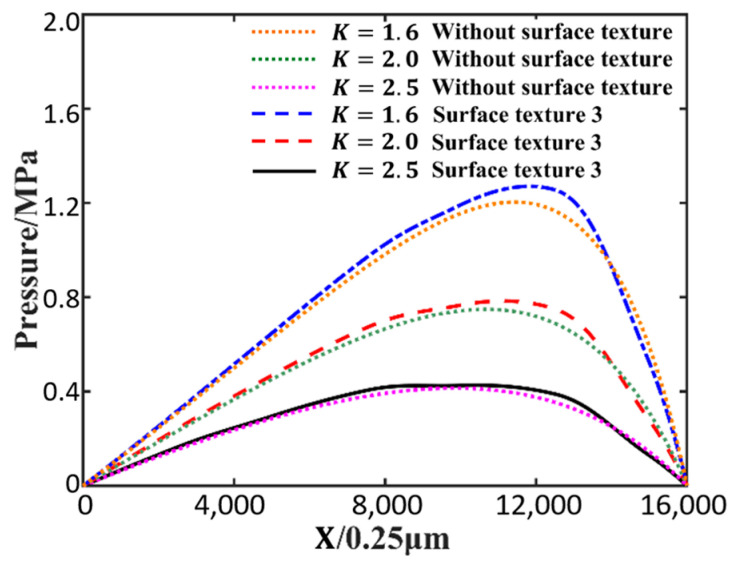
Effect of parameter K on oil film pressure.

In order to compare the effect of the parameter K on the oil film pressure with surface texture 3 and without surface texture, the inlet oil film thickness $h_{1}\;\mathrm{is}\;20\;\mu m$, and the variable is outlet oil film thickness $h_{0}=12.5\;\mu m$, 10 μm and 8 μm. The corresponding parameter K becomes 1.6, 2.0 and 2.5. Figure 14 shows the effect of different parameters K on the oil film pressure with and without surface texture. Under the condition that the ratio of inlet and outlet oil film thickness *K* is changed, the oil film pressure changes of surface texture 3 and no surface texture are shown in Figure 14. The results show that the effect of surface texture on oil film pressure is greater when the ratio of inlet and outlet oil film thickness is larger. When the ratio of inlet and outlet oil film thickness is 2.5, the effect of surface texture on oil film pressure is greater. The maximum oil film pressure increases by 5.56% compared to without surface texture. The maximum oil film pressure increased by 4.51% and 2.96% when the ratio of inlet to outlet film thickness was 2.0 and 1.6, respectively.

**Figure 15:**
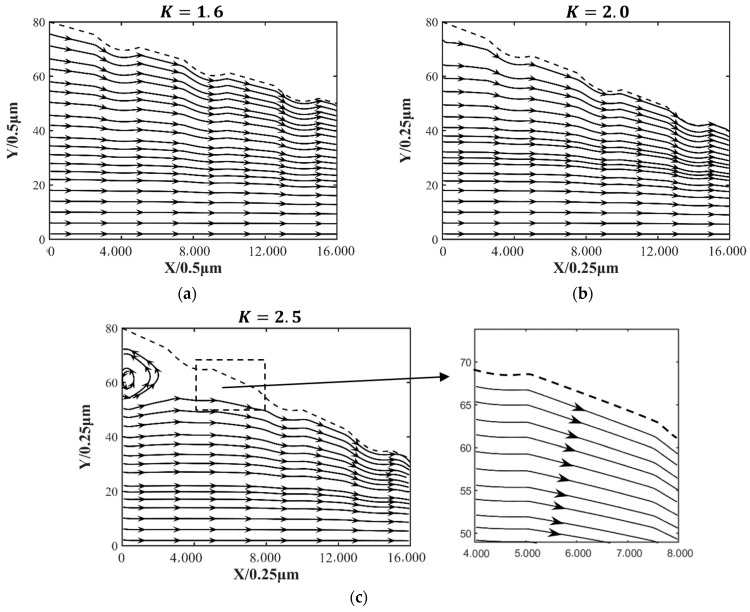
The streamlines pattern of the oil film. (**a**) Streamline pattern for the parameter K=1.6; (**b**) streamline pattern for the parameter K=2.0; (**c**) streamline pattern for the parameter K=2.5. The dashed line is the slider profile.

Figure 15 shows the streamline pattern for the different parameter K. The calculation results show that the presence of a vortex is found at the inlet position of the oil film when the parameter K=2.5. As the parameter K increases, the more likely a vortex is formed at the inlet position of the oil film. As *K* increases, the surface angle of the wedge-shaped slider becomes larger, and at the same time, there are convex shapes on the surface, which hinders the fluid greatly, so a vortex is generated at the entrance. According to Figure 15c, it can be seen that after the first vortex is generated, the flow field is not exactly the same as the flow field at the entrance, and no vortex is generated at a downstream location.

In this paper, only the influence of surface micro-texture of one shape on lubrication is discussed. Later studies can explore the influence of the shape, distribution and density of micro-texture on lubrication.

## 6. Conclusions

In this paper, the effect of surface textures on micro lubrication and heat transfer is calculated using the incompressible double distribution LBM, which gives the velocity distribution and temperature distribution in the direction of oil film thickness. There are several conclusions, given as follows.

(1) Under the same conditions, comparing the oil film pressure with and without surface texture, the results show that the maximum oil film pressure with surface texture 3 is increased by about 4.34% compared with that without surface texture. The micro convex texture can increase the hydrodynamic lubrication effect and obtain greater load-bearing capacity, helping to reduce the possibility of contact friction.
(2) At 3.6 mm in the slider length direction and 7.5 μm in the oil film thickness direction, the temperature of surface texture 1 is 402.64 K, the temperature of surface texture 2 is 403.31 K, and the temperature of surface texture 3 is 403.99 K. The convex surface texture can increase the heat transfer area and reduce distance to the bottom boundary, which can increase the effect of heat conduction. This may provide the basis for heat dissipation from lubrication and consideration of the rheological effects of the lubricant.
(3) Compared with the Reynolds equation, this method allows us to solve the velocity distribution of the oil film, and the occurrence of a vortex was observed at a ratio of inlet and outlet oil film thickness of 2.5, allowing more microscopic details to be observed.
(4) With a low ratio of inlet and outlet oil film thickness, the micro convex surface texture has a limited effect on the oil film pressure. However, the physical model proposed in this article is only 4 mm. If applied to a large geometric area, it will generate a huge number of calculations, and it is only applicable to situations where the Reynolds number is low.

## Figures and Tables

**Table 1:** Lubricant data.

Parameters	Values
Lubricant density	855 kg/m^3^
Dynamic viscosity	0.0027 Pa·s
Thermal conductivity	0.141 W/(m·K)
Specific heat capacity	2088 J/(kg·K)
Prandtl number	40

**Table 2:** Parameters of the slider.

Parameter	Value
Wedge length	4 mm
Inlet film thickness	20 μm
Outlet film thickness	10 μm
Velocity of bottom boundary	4 m/s
Dynamic viscosity	0.0027 Pa·s
Density	855 kg/m^3^
Prandtl number	40
Number of lattice N_x_ × N_y_	16,000 × 80

**Table 3:** Parameters of surface texture.

Surface Texture	Surface Texture 1	Surface Texture 2	Surface Texture 3
A	0.25 μm	0.5 μm	0.75 μm
ω	0.0004 × π	0.0004 × π	0.0004 × π

## Data Availability

Data are contained within the article.

## List of Symbols

u	speed
$h_{1}$	oil film thickness of the left inlet
$h_{0}$	oil film thickness of the right outlet
L	length of the wedge
K	ratio of inlet and outlet oil film thickness
A	amplitude
ω	scaling factor of the transverse coordinate
$\tau_{f},\tau_{g}$	relaxation time
υ	kinetic viscosity
$e_{i}$	particle motion velocity
$f_{i}^{eq}$	equilibrium distribution function
ρ	macroscopic volume density
$w_{i}$	weight factor
c	lattice velocity
p	oil film pressure
μ	dynamic viscosity
$\rho_{0}$	initial fluid density
$g_{i}$	distribution function of the temperature field
α	thermal diffusion coefficient
$g_{i}^{eq}$	equilibrium distribution function of the temperature field
T	fluid temperature
q	percentage of fluid area
$f_{i}^{neq}$	nonequilibrium distribution function
*N_x_*, *N_y_*	number of lattice
l	characteristic length

## References

[1] Nsilani Kouediatouka A., Ma Q., Liu Q., Mawignon F.J., Rafique F., Dong G. (2022). Design methodology and application of surface texture: A review. Coatings.

[2] Zhang H., Zhang D., Hua M., Dong G., Chin K. (2014). A study on the tribological behavior of surface texturing on babbitt alloy under mixed or starved lubrication. Tribol. Lett..

[3] Li P., Zhang F., Zhang H., Wang T., Wang Q., Qiao W. (2023). Lubrication performance of kite-shaped microtexture under hydrodynamic lubrication. Tribol. Int..

[4] Wang Y., Zhou T., Riemer O., Heidhoff J., Li M., Karpuschewski B., Gorb N., Schaber C. (2022). Tribological mechanism of micro/meso/macroscopic textured surfaces under different normal forces, relative velocities, and sliding directions. Tribol. Int..

[5] Shen Z., Wang F., Chen Z., Ruan X., Zeng H., Wang J., An Y., Fan X. (2021). Numerical simulation of lubrication performance on chevron textured surface under hydrodynamic lubrication. Tribol. Int..

[6] Guo Z., Xie X., Yuan C., Bai X. (2019). Study on influence of micro convex textures on tribological performances of UHMWPE material under the water-lubricated conditions. Wear.

[7] Gu C., Meng X., Xie Y., Fan J. (2016). A thermal mixed lubrication model to study the textured ring/liner conjunction. Tribol. Int..

[8] Pei S., Xu H., Yun M., Shi F., Hong J. (2016). Effects of surface texture on the lubrication performance of the floating ring bearing. Tribol. Int..

[9] Ruan J., Wang X., Wang Y., Li C. (2022). Study on anti-scuffing load-bearing thermoelastic lubricating properties of meshing gears with contact interface micro-texture morphology. J. Tribol..

[10] Heredia-Cancino J., Ramezani M., Álvarez-Ramos M. (2018). Effect of degradation on tribological performance of engine lubricants at elevated temperatures. Tribol. Int..

[11] Guan L., Feng X., Xiong G., Xie J. (2011). Application of dielectric spectroscopy for engine lubricating oil degradation monitoring. Sens. Actuator A-Phys..

[12] Harigaya Y., Suzuki M., Toda F., Takiguchi M. (2006). Analysis of oil film thickness and heat transfer on a piston ring of a diesel engine: Effect of lubricant viscosity. J. Eng. Gas. Turbines Power-Trans. ASME.

[13] Felter C.L. (2008). Numerical simulation of piston ring lubrication. Tribol. Trans..

[14] Sahlin F., Glavatskih S.B., Almqvist T.R., Larsson R. (2005). Two-dimensional CFD-analysis of micro-patterned surfaces in hydrodynamic lubrication. J. Trib..

[15] Shyu S.-H., Hsu W.-C. (2018). A numerical study on the negligibility of cross-film pressure variation in infinitely wide plane slider bearing, Rayleigh step bearing and micro-grooved parallel slider bearing. Int. J. Mech. Sci..

[16] Abadshapoori M.H., Saidi M.H. (2018). 3D investigation of natural convection of nanofluids in a curved boundary enclosure applying lattice Boltzmann method. Int. J. Numer. Methods Heat Fluid Flow.

[17] Chen S., Doolen G.D. (1998). Lattice Boltzmann method for fluid flows. Annu. Rev. Fluid Mech..

[18] Chen H.-T., Lin J.-Y. (1993). Numerical analysis for hyperbolic heat conduction. Int. J. Heat Mass Transf..

[19] Gupta N., Chaitanya G.R., Mishra S.C. (2006). Lattice Boltzmann method applied to variable thermal conductivity conduction and radiation problems. J. Thermophys. Heat Transf..

[20] Brenner G., Al-Zoubi A., Mukinovic M., Schwarze H., Swoboda S. (2007). Numerical simulation of surface roughness effects in laminar lubrication using the lattice-Boltzmann method. J. Tribol.-Trans. ASME.

[21] Jiao C., Leng Z., Zou D., Ta N., Rao Z. (2021). Numerical research of the infinitely wide wedge flow based on the lattice Boltzmann method. Proc. Inst. Mech. Eng. Part J.-J. Eng. Tribol..

[22] Yagi K., Sugimura J. (2013). Balancing Wedge Action: A Contribution of Textured Surface to Hydrodynamic Pressure Generation. Tribol. Lett..

[23] Zhang Y., Li H.N., Li C., Huang C., Ali H.M., Xu X., Mao C., Ding W., Cui X., Yang M. (2022). Nano-enhanced biolubricant in sustainable manufacturing: From processability to mechanisms. Friction.

[24] Xin C., Changhe L., Wenfeng D., Yun C., Cong M., Xuefeng X., Bo L., Dazhong W., Li H.N., Zhang Y. (2022). Minimum quantity lubrication machining of aeronautical materials using carbon group nanolubricant: From mechanisms to application. Chin. J. Aeronaut..

[25] Cui X., Li C., Zhang Y., Ding W., An Q., Liu B., Li H.N., Said Z., Sharma S., Li R. (2023). A comparative assessment of force, temperature and wheel wear in sustainable grinding aerospace alloy using bio-lubricant. Front. Mech. Eng..

[26] Cui X., Li C., Zhang Y., Said Z., Debnath S., Sharma S., Ali H.M., Yang M., Gao T., Li R. (2022). Grindability of titanium alloy using cryogenic nanolubricant minimum quantity lubrication. J. Manuf. Process..

[27] Xu B., Lu X., Jiang Y., Xiong C., Yu H., Luo X., Chen Z. (2022). Research on clearance flow characteristics of gas-lubricated journal bearings using the lattice Boltzmann method. Alex. Eng. J..

[28] He X., Luo L.-S. (1997). Theory of the lattice Boltzmann method: From the Boltzmann equation to the lattice Boltzmann equation. Phys. Rev. E.

[29] Aidun C.K., Clausen J.R. (2010). Lattice-Boltzmann method for complex flows. Annu. Rev. Fluid Mech..

[30] Perumal D.A., Dass A.K. (2015). A Review on the development of lattice Boltzmann computation of macro fluid flows and heat transfer. Alex. Eng. J..

[31] Degrigny J., Cai S.-G., Boussuge J.-F., Sagaut P. (2021). Improved wall model treatment for aerodynamic flows in LBM. Comput. Fluids.

[32] Bhatnagar P.L., Gross E.P., Krook M. (1954). A model for collision processes in gases. I. Small amplitude processes in charged and neutral one-component systems. Phys. Rev..

[33] He X., Luo L.-S. (1997). Lattice Boltzmann model for the incompressible Navier–Stokes equation. J. Stat. Phys..

[34] Sharma K.V., Straka R., Tavares F.W. (2020). Current status of Lattice Boltzmann Methods applied to aerodynamic, aeroacoustic, and thermal flows. Prog. Aeosp. Sci..

[35] Kefayati G. (2014). Natural convection of ferrofluid in a linearly heated cavity utilizing LBM. J. Mol. Liq..

[36] Zhao-Li G., Chu-Guang Z., Bao-Chang S. (2002). Non-equilibrium extrapolation method for velocity and pressure boundary conditions in the lattice Boltzmann method. Chin. Phys..

[37] Guo Z., Zheng C., Shi B. (2002). An extrapolation method for boundary conditions in lattice Boltzmann method. Phys. Fluids.

[38] Yan Y., Zu Y. (2008). Numerical simulation of heat transfer and fluid flow past a rotating isothermal cylinder—A LBM approach. Int. J. Heat Mass Transf..

[39] Sheikholeslami M., Gorji-Bandpy M., Vajravelu K. (2015). Lattice Boltzmann simulation of magnetohydrodynamic natural convection heat transfer of Al_2_O_3_–water nanofluid in a horizontal cylindrical enclosure with an inner triangular cylinder. Int. J. Heat Mass Transf..

